# Theranostic gold-in-gold cage nanoparticles enable photothermal ablation and photoacoustic imaging in biofilm-associated infection models

**DOI:** 10.1172/JCI168485

**Published:** 2023-11-01

**Authors:** Maryam Hajfathalian, Christiaan R. de Vries, Jessica C. Hsu, Ahmad Amirshaghaghi, Yuxi C. Dong, Zhi Ren, Yuan Liu, Yue Huang, Yong Li, Simon A.B. Knight, Pallavi Jonnalagadda, Aimen Zlitni, Elizabeth A. Grice, Paul L. Bollyky, Hyun Koo, David P. Cormode

**Affiliations:** 1 Department of Radiology, University of Pennsylvania, Philadelphia, Pennsylvania, USA.; 2 Division of Infectious Diseases, School of Medicine, Stanford University, Stanford, California, USA.; 3 Department of Bioengineering,; 4 Department of Orthodontics and Divisions of Pediatric Dentistry & Community Oral Health, School of Dental Medicine, and; 5 Department of Dermatology and Microbiology, Perelman School of Medicine, University of Pennsylvania, Philadelphia, Pennsylvania, USA.; 6 Department of Radiology, School of Medicine, Stanford University, Stanford, California, USA.

**Keywords:** Infectious disease, Therapeutics, Bacterial infections

## Abstract

Biofilms are structured communities of microbial cells embedded in a self-produced matrix of extracellular polymeric substances. Biofilms are associated with many health issues in humans, including chronic wound infections and tooth decay. Current antimicrobials are often incapable of disrupting the polymeric biofilm matrix and reaching the bacteria within. Alternative approaches are needed. Here, we described a complex structure of a dextran-coated gold-in-gold cage nanoparticle that enabled photoacoustic and photothermal properties for biofilm detection and treatment. Activation of these nanoparticles with a near infrared laser could selectively detect and kill biofilm bacteria with precise spatial control and in a short timeframe. We observed a strong biocidal effect against both *Streptococcus mutans* and *Staphylococcus aureus* biofilms in mouse models of oral plaque and wound infections, respectively. These effects were over 100 times greater than those seen with chlorhexidine, a conventional antimicrobial agent. Moreover, this approach did not adversely affect surrounding tissues. We concluded that photothermal ablation using theranostic nanoparticles is a rapid, precise, and nontoxic method to detect and treat biofilm-associated infections.

## Graphical abstract

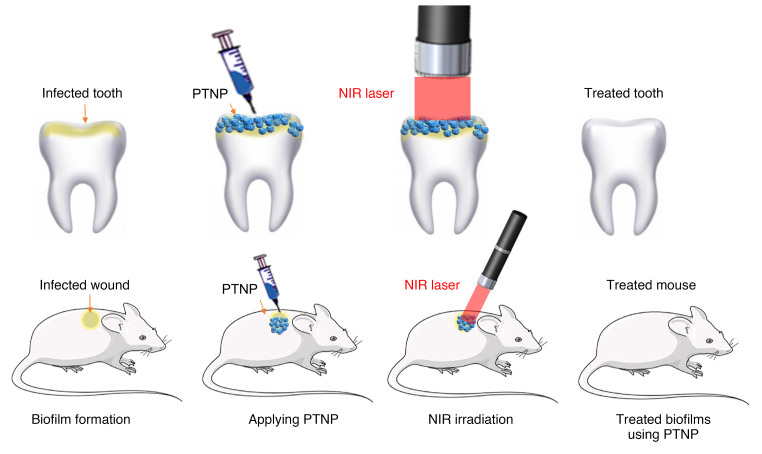

## Introduction

Biofilms, communities of bacteria within an extracellular polymeric substance (EPS), are implicated in many chronic infections (1–3). Once biofilms are established, they are notoriously difficult to eradicate due to the presence of an extracellular matrix surrounding the bacteria and protecting them from antibiotics (4, 5). Hence, it is important to locate the biofilms for rapid and precise action against them. Current therapeutic modalities struggle to disrupt biofilms and often fail to efficiently kill the microbes within (6, 7). Consequently, biofilm-associated infections are responsible for human suffering and massive economic expense (8). The development of therapies capable of killing biofilm-associated bacteria would have a significant impact on the treatment of chronic infections.

Two locations where biofilms are relevant to human health are the skin and the mouth. Dental caries afflict nearly half of the world’s population and are a major public health problem according to the US Centers for Disease Control (9). One of the major oral pathogens involved in dental caries is *Streptococcus mutans* (*S*. *mutans*); its ability to form biofilms is a key aspect of this pathology (10, 11). Over the past decade researchers have devised innovative antimicrobial strategies to inhibit *S*. *mutans* biofilm formation (12–14). Unfortunately, antibacterial resistance, a lack of appropriate diagnostic tools, and a paucity of convenient and affordable treatments remain barriers to treating oral biofilms (15, 16).

Wound infections affect 6.5 million people in the USA, with estimated annual costs of $28 billion (17). Bacterial biofilms are a major factor in wound chronicity due to effects on delayed healing and impaired bacterial clearance by antibiotics. In most contexts, *Staphylococcus* species are the most common bacteria recovered from chronic wounds. There are many strategies to clear wound biofilms, with periodic wound debridement being the most common (18, 19). However, wound biofilms remain a major unsolved and growing problem (20).

Photothermal therapy (PTT) is an antibacterial strategy that utilizes light sources to activate nanoagents and convert the light energy into heat, killing the pathogens (21, 22). This antibacterial strategy is noninvasive and effective against biofilm bacteria, including those resistant to conventional antibiotic drugs (23–25). However, there is still a need to develop antibiofilm agents that are photothermally efficient, biocompatible, and photostable (26–28). Advances in shape engineering, synthesis methods, and coatings of nanoparticles and their combinations with organic materials have introduced new functionalities for nanostructures and their utility in different bioapplications and therapeutic methods (29, 30). Gold nanoparticles, in particular, have been used to enhance antibiotic efficacy and reduce bacterial viability in vitro (31–33). Optically tuned gold nanoparticles with different sizes and structures have been studied for their use in photothermal ablation (34, 35). Gold nanorods, nanostars, and nanoshells have shown strong antibacterial effects against bacterial biofilms (36–38). In addition, gold nanocages with a hollow interior and porous walls have been used as PTT agents (39–41). However, these nanoparticles need to be engineered to have a strong extinction spectrum in the near infrared (NIR) region and higher photothermal efficiency to be capable of killing bacteria effectively and treating infections in a short time frame. Moreover, preclinical studies (specifically for oral infections) are lacking most of these agents. There is an urgent need for ex vivo and in vivo demonstrations of efficacy that would allow further development of PTT (14, 42).

There is also a need to visualize biofilm infections on wounds, teeth, and gums in order to define the infected lesions precisely, determine the depth, and document ablation in real time. Photoacoustic imaging (PA) is a powerful imaging technique that allows detection of bacterial infections (43, 44). PA utilizes photothermal expansion of light, absorbing contrast agents to generate ultrasound waves under pulsed laser irradiation. PA has less background, higher spatial resolution, stronger contrast, and better deep tissue penetration than optical imaging (45, 46). The development of a multi-functional platform that could image oral and wound bacterial infections and ablate bacterial infections simultaneously would be a considerable advantage to infection control efforts.

Here, we report multifunctional gold-in-gold cage photothermal nanoparticles (PTNP), which specifically detect biofilm and kill bacteria with high spatial precision in less than a minute. These nanoparticles were formed by growth of a silver shell over a gold core and subsequent galvanic replacement of the silver with a gold cage. This morphology has a plasmon peak in NIR that provides them an excellent performance in biomedical applications involving optical excitation or transduction. We have evaluated the photothermal properties, biofilm uptake, antimicrobial action, cytotoxicity, and PA ability of these nanoparticles. Our in vitro data demonstrate high potency to eradicate biofilms and eliminate wound and oral pathogens without toxicity to eukaryotic cells. Furthermore, PTNP was examined for its efficacy as an antibiofilm agent to detect and treat in vivo biofilms in wound infections and on teeth using rodent models. These findings demonstrate the potential of these nanostructures for therapeutic applications.

## Results

### Synthesis of gold-in-gold cage photothermal nanoparticles

**Figure 1:**
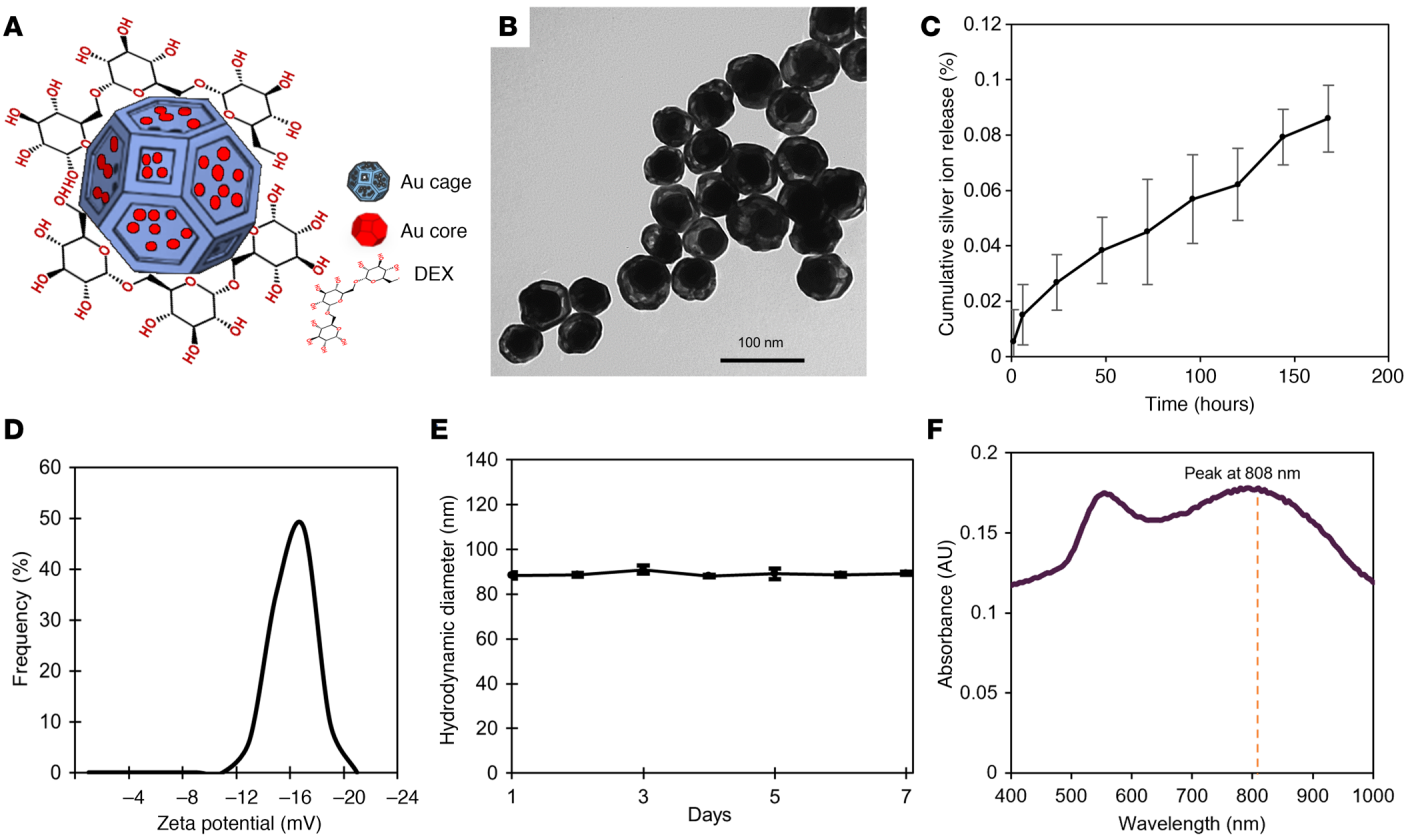
PTNP with complex morphologies are stable and alternative plasmonic nanoagents for biomedical applications. (**A**) Schematic depiction of PTNP structure, which consists of an Au core encapsulated by Au cage and coated with DEX. (**B**) TEM of PTNP (Scale bar: 100 nm). (**C**) Silver ion release from PTNP when incubated in DI water at 37°C over a period of 7 days (*n* = 6). (**D**) ζ Potential data of DEX-coated PTNP. (**E**) Hydrodynamic diameter of PTNP in DI water during a week (*n* = 4). (**F**) UV-vis spectrum of DEX-PTNP. The data are presented as mean ± SD.

Gold-in-gold cage nanoparticles were synthesized through the following method: a modified Turkevich method was used to develop small seeds (47). A seeded growth method was then used to make larger nanoparticles with the equilibrium shape — a truncated octahedron enclosed by 6 square facets and 8 hexagonal facets (48) — which we term cores in this manuscript. We then performed a reduction of silver nitrate (AgNO_3_) onto the cores to produce core in a shell structures followed by a galvanic replacement reaction (GRR) of silver with gold ions (HAuCl_4_) to change the shape of the shells into nanocages, which we termed ‘gold-in-gold cage’. Figure 1, A and B show a schematic depiction and transmission electron microscopy (TEM) image of the PTNP, respectively.

For this experiment, the small gold nanoparticles (Au seeds,16 ± 2 nm) acted as nucleation sites and a seeded-growth synthesis method was used to synthesize the larger seeds (Au core, 78 ± 3 nm). Then, AgNO_3_ was reduced with ascorbic acid (AA) in a solution of Au cores to make a core-shell structure (Au-Ag, 86 ± 6 nm) (Supplemental Figure 1; supplemental material available online with this article; https://doi.org/10.1172/JCI168485DS1). The thickness of these shells was measured using TEM and was found to be 8 ± 1 nm. The size of these is tunable based on the reaction time, the amount of reducing agent, and the concentration of AgNO_3_. The next step of the shape engineering to create a gold-in-gold cage structure was adding HAuCl_4_ to the Au-Ag structure solution to initiate GRR, which is a spontaneous oxidation–reduction reaction where HAuCl_4_ is reduced and silver is oxidized. This reaction caused the formation of cages with a size of 85 ± 5 nm around the Au cores. Finally, dextran (DEX) coating was added to stabilize the cage nanoparticles and enhance their biocompatibility and uptake by biofilms. In this manuscript, PTNP refers to DEX-coated gold-in-gold cage nanoparticle. The composition of PTNP is 87 ± 2% Au and 13 ± 4% Ag, as measured by inductively coupled plasma–optical emission spectroscopy (ICP-OES) analysis. These results were also confirmed with energy dispersive X-ray spectroscopy (EDS) measurements (Supplemental Figure 2A). The low amount of silver on cages as well as its being alloyed with Au can be expected to suppress possible silver ion release, improve the overall stability, and extend the lifetime of the antibiofilm agent. Figure 1C shows that silver ion release from PTNP over 7 days is very low (less than 0.085%), and it is much lower than that found for pure silver nanoparticles (49). This result agrees with prior work that showed that alloying silver with gold suppressed silver ion release (50).

The ζ potential of PTNP was found to be –17 ± 1.5 mV, similar to other DEX-coated nanoparticles (Figure 1D) (51, 52). The hydrodynamic diameters of nanoparticles were found to be larger than the diameters measured from TEM images due to coating and hydration layers (Supplemental Figure 2B), however the difference was not statistically significant (53). The hydrodynamic diameters of gold seeds, gold cores, core-shells and PTNP were found to be 18.2 ± 0.2, 79.2 ± 1.1 nm, 88.1 ± 2.2, and 87.7 ± 1.4 nm, respectively. In this study, PTNP were stable and no change in color was observed after 15 days’ incubation of nanoparticles with media, PBS, or saliva (Supplemental Figure 3). Figure 1E shows the size distribution of PTNP measured with DLS for a week, which does not change over time, confirming the stability of these structures in deionized (DI) water.

### Optical behavior

Spherical gold nanoparticles (AuNPs) produce plasmonic properties (40, 53), but they do not provide strong absorption spectra in NIR (Supplemental Figure 4). Plasmonic properties in NIR are needed to make them an effective candidate in biomedical applications (54). Therefore, engineering AuNPs characteristics such as shape, size, and composition could help to establish a new agent with the specific purpose of biofilm treatment methods. The plasmonic properties of shape-engineered gold-in-gold cage nanoparticle were measured using UV-vis spectroscopy and are shown in Figure 1F. These nanoparticles have a plasmon peak at 808 nm which is tunable in the NIR region based on its GRR and the thickness of shells (55) and make them a great candidate for biomedical applications.

### PTNP incorporation within biofilms

**Figure 2:**
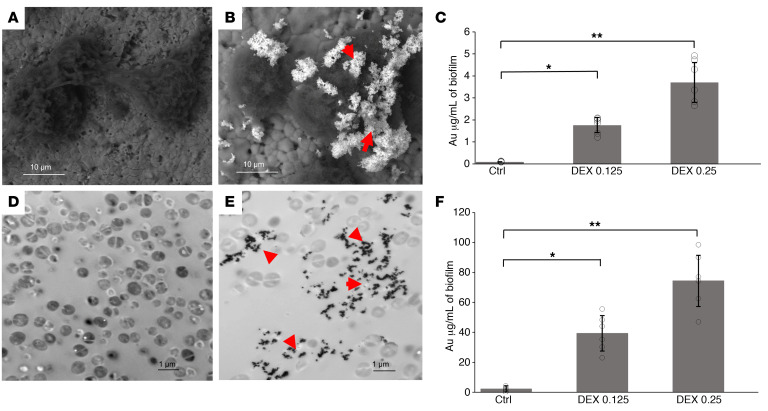
PTNP are taken up by bacterial biofilms. SEM in backscattered electron (BSE) mode showing the morphology of (**A**) untreated biofilm, (**B**) PTNP-treated *S*. *mutans* biofilm (Scale bar: 10 μm). Red arrows show nanoparticles. (**C**) ICP-OES measurements on *S*. *mutans* biofilms. TEM of *S*. *aureus* biofilm uptake of PTNP, (**D**) untreated biofilm, and (**E**) *S*. *aureus* biofilm treated with PTNP for 24 hours (Scale bar: 1 μm). (**F**) ICP-OES measurements on *S*. *aureus* biofilms, (*n* = 6). Red arrows show nanoparticles. Statistical analyses used a mixed-model ANCOVA with SAS 9.5 software from SAS Institute. *P < 0.05, **P < 0.005. The data are presented as mean ± SD.

To measure the incorporation of PTNP within biofilm, different concentrations of PTNP (0, 0.1, and 0.25 mg/mL) were coated with 3 different coatings, i.e., polyethylene glycol (PEG), DEX-10kDa and polydopamine (PDA). Supplemental Figure 5 shows DEX-enhanced incorporation of PTNP into *S*. *mutans* biofilms much more than PDA and PEG. We found that PTNP were incorporated into *S*. *mutans* and *S*. *aureus* biofilms while using a low concentration of PTNP (0.25 mg/mL). SEM images of biofilms incubated with PTNP indicated that PTNP bind to *S*. *mutans* biofilms (Figure 2, A and B), which was confirmed by EDS measurements (Supplemental Figure 6, A–C). To investigate PTNP binding in biofilms of *S*. *aureus* we also carried out TEM of biofilms (Figure 2, D and E), which showed clear uptake and penetration of PTNP within the biofilm ultrastructure. Figure 2, C and F show that PTNP are significantly taken up into *S*. *mutans* and *S*. *aureus* biofilms, as measured by inductively coupled plasma optical emission spectroscopy (ICP-OES). These data confirm DEX-enhanced incorporation of PTNP into *S*. *mutans* and *S*. *aureus* biofilms, while no binding enhancement was observed on our control sample, a saliva-coated apatite surface (sHA). This polymer promotes selectivity toward biofilms while avoiding binding to host cells. DEX is a component of biofilms and prior evidence suggests that DEX-coated nanoparticles are incorporated into biofilms by the enzyme β-glucosyltransferase (gtfB) (4, 52). These results demonstrate the successful incorporation of PTNP into *S*. *aureus* biofilms when applied topically, supporting the idea of using PTNP for PA and PTT of biofilms.

### Photothermal activity of PTNP

**Figure 3:**
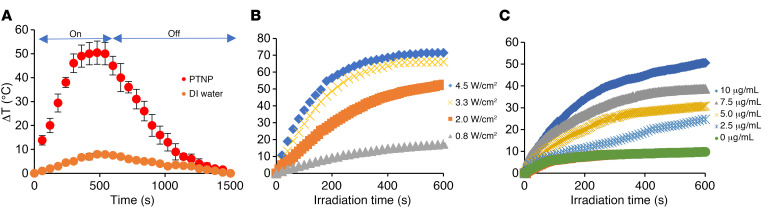
PTNP have excellent photothermal properties. (**A**) Heating and cooling curves of PTNP (10 μg/mL) and DI water. (**B**) Temperature changes of solutions containing 10 μg/mL PTNP irradiated at laser power densities of 0.8, 2, 3.3, or 4.5 W/cm^2^. (**C**) Temperature changes of solutions containing 0, 2.5, 5, 7.5, or 10 μg/mL PTNP irradiated at a laser power density of 2 W/cm^2^ (*n* = 5). Statistical analyses used a mixed-model ANCOVA with SAS 9.5 software from SAS Institute. The data are presented as mean ± SD.

Next, we examined the heat generation of PTNP while irradiated with a NIR laser. PTNP solutions and DI water as a control solution were treated with a NIR laser, and the temperature was recorded over time. PTNP structures were found to generate significant temperature increases, higher than the control solution. Figure 3A shows the maximum temperature observed for the PTNP solution was much higher than that for DI water (as much as 50°C). The photothermal conversion efficiency of PTNP was calculated to be 77%, which is comparable with gold nanostars (78%) (56) and gold nanoporous nanoshells (75.5%) (57), and higher than gold nanocages (53.6%) (58, 59), gold nanorings (42%) (60), gold nanoshells (41.4%) (61), and gold nanorods (21.3%) (61, 62). The temperature change of the PTNP and its dependency on laser power and PTNP concentration was also studied. As is shown in Figure 3B, greater laser power density resulted in greater temperature increases. Similarly, increases in the concentration of PTNP led to greater temperature enhancement (Figure 3C).

We found that during 5 cycles of laser on-off irradiation, the PTNP solution reproducibly reached 99.9% of the temperature achieved the first time, indicating the excellent stability of these structures (Supplemental Figure 7A). The TEM of irradiated PTNP samples did not show any difference in morphology, which confirms the photostability of these structures (Supplemental Figure 7, B and C). Here, we chose different laser powers depending on the experiment setting and in agreement with the work of others (60, 63, 64) For only in vitro and ex vivo oral biofilms that develop upon hard surfaces such as hydroxyapatite discs or teeth, we used higher laser power (2 W/cm^2^) and shorter time frames, which is, again, in agreement with recent studies that report powers of 2–2.5 W/cm^2^ (64, 65). For in vitro and in vivo skin studies we used lower laser powers (0.25–0.7 W/cm^2^) to avoid any possible damage in surrounding tissues, as has been reported in other studies that use powers such as 0.75, 0.78, and 1 W/cm^2^ (56, 66, 67).

To test the biofilm imaging ability of PTNP, we examined the PA signal of PTNP at different PTNP concentrations (0, 0.06, 0.12, 0.25, and 0.5 mg/mL). Increasing PA signal was observed with increasing the concentration of PTNP (Supplemental Figure 8, A and B). We also examined the PA properties of PTNP when incubated with *S*. *aureus* biofilm. The PA signal was assessed using different PTNP concentrations (0, 0.12, 0.25, and 0.5 mg/mL) which were incubated with biofilm for 24 hours and were imaged at a laser wavelength of 800 nm (Supplemental Figure 9). The PA signal rose with increases in the concentration of PTNP. These findings suggest that PTNP can be used as an agent for both diagnosis and treatment of oral and skin infectious diseases.

### In vitro antibiofilm efficacy of the PTNP

**Figure 4:**
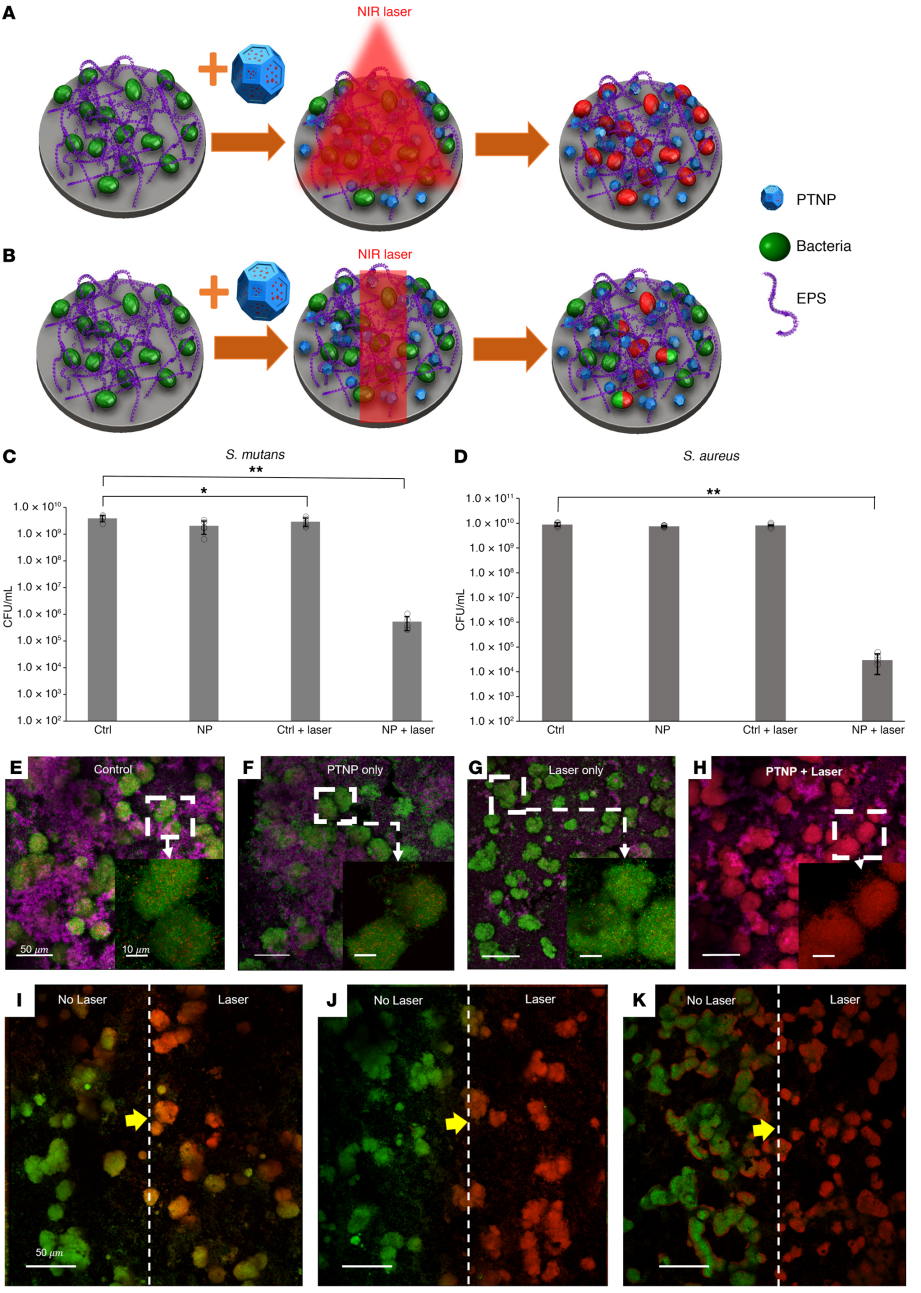
PTNP efficiently kill biofilm bacteria in vitro. (**A**) Schematic of photo ablation of biofilm with PTNP in wide area mode and (**B**) precise spatial control mode. The viability of (**C**) *S*. *mutans* and (**D**) *S*. *aureus* within biofilms treated with PTNP and irradiated in wide area mode (*n* = 5). The statistical technique used in this experiment is a 2-way ANOVA with Šidák’s multiple comparison correction. *P < 0.05, **P < 0.0001. (**E**–**H**) Representative confocal micrographs of treated and untreated *S*. *mutans* biofilms exposed to wide area mode (Scale bar: 50 μm). Magnified views of *S*. *mutans* biofilms in the boxed areas are shown in the bottom left of each panel (Scale bar: 10 μm). Confocal micrograph images of PTNP-treated *S*. *mutans* biofilms exposed to laser in precise control mode for (**I**) 15 seconds, (**J**) 30 seconds, and (**K**) 60 seconds. Live bacteria cells (green), dead cells (red), and EPS (purple) are shown (Scale bar: 50 μm). Biofilm bacteria were irradiated at 808 nm (**C**–**H** and **I**–**K** were irradiated with laser power 0.5 W/cm^2^ and 2 W/cm^2^, respectively).

Here, we examined how PTNP combined with laser irradiation disrupts biofilms and kills bacteria. Oral biofilms (*S*. *mutans* UA159, a strain associated with endocarditis) were formed on sHA discs, while *S*. *aureus* biofilms were grown in 96-wall plates as a skin infection model. Biofilms were analyzed using multiphoton confocal microscopy, colony counting (presented as colony forming units or CFU), and computational analysis (COMSTAT and Image J). Biofilms treated with or without PTNP (0.25 mg/mL) were exposed to NIR laser irradiation at 808 nm in both wide-area and precise spatial–control irradiation mode (schematically shown in Figure 4, A and B, respectively), at 0.5 and 2 W/cm^2^ for different time points. Then, bacterial survival studies were conducted on PTNP surfaces using a colony formation assay both with and without NIR irradiation in a wide area mode. The biofilms were removed, homogenized, and the number of viable cells were determined.

We observed an exceptionally strong biocidal effect against *S*. *mutans* and *S*. *aureus* within biofilms when exposed to NIR laser irradiation and PTNP, causing a greater-than 5-log and greater-than 6-log reduction (elimination of 99.99% of bacteria) of viable oral and skin pathogens, respectively (Figure 4, C and D). This treatment is considerably more effective than the current clinically used oral and skin antimicrobials such as chlorhexidine, alcoholic chlorhexidine, and 0.5% chlorhexidine-10% povidone iodine, which causes around 3-log reduction in the same conditions (68–70). We observed that the dry weight of remaining *S*. *mutans* biofilms in the treated samples with NIR light was 41.1% less than the control samples and untreated samples (Supplemental Figure 10). This was surprising, given the short laser irradiation time of this procedure. These results suggest that the treatment can cause EPS degradation as well as killing of the bacteria. These studies demonstrated that PTNP, when illuminated with NIR light, could effectively kill *S*. *mutans* and *S*. *aureus*, as well as degrade biofilm EPS.

### The effects of PTNP and laser treatment at a local and wide area level

To further understand how PTNP can be used as a biofilm treatment, we performed treatment experiments using confocal microscopy. The same treatment procedure was repeated (irradiating the samples with NIR laser in wide area mode, schematic is shown in Figure 4A) in the last section. Representative images of control, PTNP-treated biofilms, exposed control biofilm, and PTNP-treated biofilms to the NIR laser are shown in Figure 4, E–H. We observed almost complete bacteria killing in biofilms incubated with PTNP and after 10 minutes of wide area–mode laser treatment, while few dead cells were observed in other samples. These results confirmed our hypothesis that photothermal ablation PTNP could kill *S*. *mutans* bacteria.

To underscore the potential targeted approach of our therapy, we used a modified version of the treatment method described above. Here, the laser beam was focused to an area in the middle of the biofilm covered disc of 2 mm × 5 mm and turned on for 15, 30, or 60 seconds (Figure 4, J and K). The untreated area is on the left and the treated area on the right in Figure 4, I–K. The 15 second NIR laser irradiation is not enough to completely kill the bacteria in the irradiated area; however, 30 seconds of irradiation can completely kill the bacteria, and there is a clear line between live and dead cells (the yellow arrows show the line). The 60 second exposure resulted in bacterial killing even in the unirradiated part of the biofilm, which indicates that, for in vitro localized treatment, 60 seconds of irradiation is too long. Here, we demonstrate how our antibiofilm agent could treat the bacteria locally in less than a minute.

### Biocompatibility and cell toxicity of PTNP

**Figure 5:**
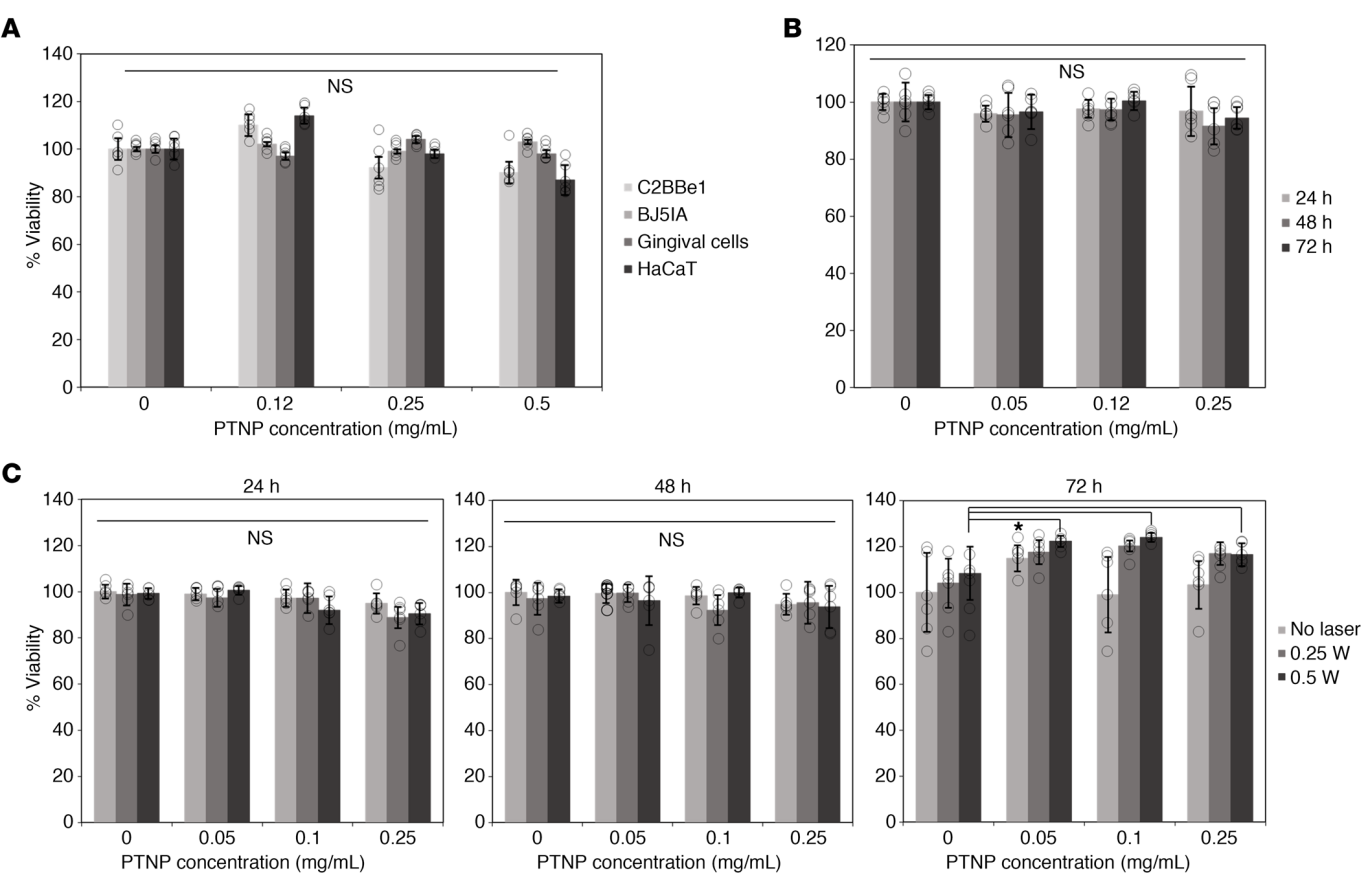
PTNP are biocompatible and nontoxic. (**A**) The effect of PTNP on the viability of several cell lines. (**B**) The cell viability of HaCaT cells in 24, 48, and 72 hours of incubation with PTNP. (**C**) Cell cytotoxicity of HaCaT cells incubated with PTNP in different concentrations (0.1, 0.05, 0.1 and 0.25 mg/mL) and time points. Cells were irradiated at 808 nm (0.25, and 0.5 W/cm^2^). Statics are a 2-tailed *t* test and mixed-model ANCOVA with SAS 9.5 software from SAS Institute. *P < 0.05. The data are presented as mean ± SD. All graphs are representative of *n* = 6 experiments.

In vitro biocompatibility and determining the photothermal activity of PTNP were carried out on different cell types. The cells were treated with DEX-coated PTNP for 4 hours at concentrations of 0.1, 0.25, and 0.5 mg/mL PTNP. The viabilities of the C2BBe1, BJ5IA, gingival, and HaCaT cell lines were determined using the MTS assay. It was found that these structures did not significantly affect the viability of these cell types and are biocompatible under the conditions tested (Figure 5A).

To evaluate the precision and selectivity of this treatment method for bacterial cells, we evaluated whether this approach could target biofilms without adversely impacting surrounding tissue. Since the amount of heat generated by PTNP can be controlled by either changing the laser power or concentration of PTNP in the medium, it can provide fine control and homogenous distribution of heat compared with conventional heating probes. For these experiments, HaCaT cells were selected and incubated with different concentrations of PTNP (0.05, 0.12, and 0.25 mg/mL) without light, and their viability were quantified after 24, 48, or 72 hours (Figure 5B). These figures confirm the biocompatibility and nontoxicity of PTNP within different cell lines. In this study, PTNP has been used as an antibiofilm for topical applications where scant uptake is to be expected, and, therefore, persistence and lack of ability to biodegrade is of lesser concern. In parallel experiments, treatment with PTNP was combined with laser irradiation at different powers (0.25 and 0.5 W/cm^2^, 7 minutes) and HaCaT cell viability was tested at 24, 48, or 72 hours after laser irradiation (Figure 5C). Live-dead staining microscopy image of HaCaT cells at 0.5 W laser irradiation at different time points is shown in Supplemental Figure 11. We found that PTNP was well-tolerated by eukaryotic cells and ineffective at killing them via laser irradiation due to lower uptake (Supplemental Figure 12) and greater robustness compared with bacteria. Moreover, the results indicate regrowth of the cells after 72 hours of laser irradiation, which shows that the effects of laser on mammalian cells were negligible, and no cell damage was observed on NIR-irradiated PTNP-treated cells at different powers of 0.25 and 0.5 W/cm^2^ for 7 minutes. This study also suggests the feasibility of carrying out photothermal antibiofilm treatment using PTNP in both oral and wound in vivo models, while keeping the surrounding tissue healthy.

### PTNP effects in animal models

#### PTNP detects and kills pathogenic oral bacteria in an animal model.

**Figure 6:**
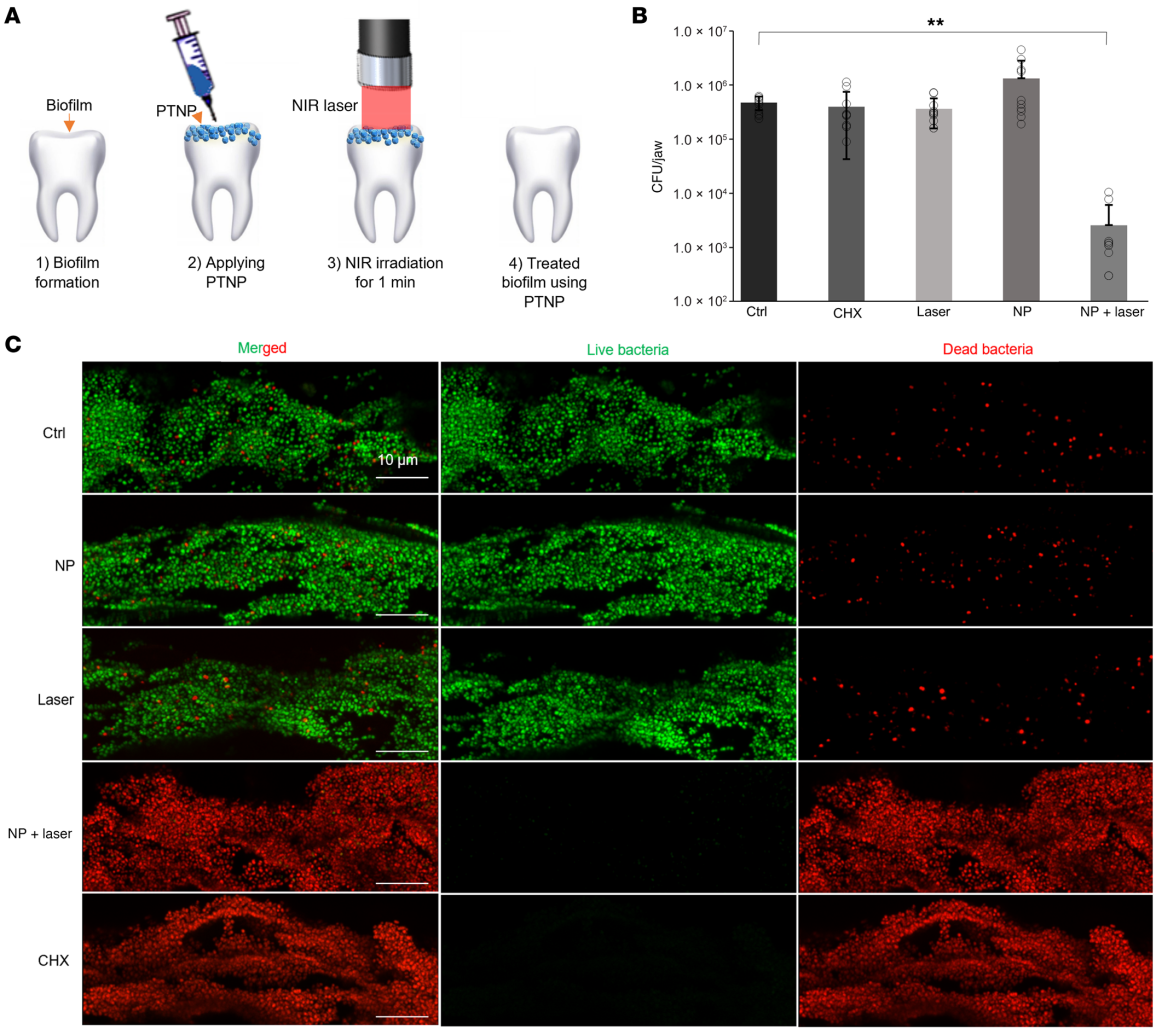
PTNP are multifunctional nanoagents that detect and treat oral infections. Photothermal efficacy of PTNP in the oral model; (**A**) A schematic of the PTT experiment. (**B**) Bacteria killing in an animal model of oral disease, as evidenced by the viability of *S*. *mutans* (*n* = 6). (**C**) Representative confocal microscopy of control, PTNP only, Laser only, PTNP,-and-laser treated, and chlorhexidine only within *S*. *mutans* biofilms. Live and dead bacteria are shown in green and red, respectively (Scale bar: 10 μm). The experimental data underwent statistical analysis using SAS 9.5 (SAS Institute) and a mixed-model ANCOVA. **P < 0.005.

The antibacterial efficacy of PTNP, with a clinically relevant topical treatment regimen for oral infections, was examined using Sprague-Dawley rats infected orally using an actively growing culture of *S*. *mutans* (71). Ex vivo PTT efficacy and PA experiment were performed on rat teeth as is shown schematically in Figure 6A. PTNP nanoparticles (0.25 mg/mL) were applied topically on the teeth, and, after 10 minutes incubation within biofilms, they were irradiated with the NIR light for 1 minute. PTNP absorbs the light and generates local high temperatures that kill *S*. *mutans* bacteria. Figure 6B shows the viability of *S*. *mutans* within PTNP-treated biofilms after the laser treatments. Here, we found that none of the other groups (control, only PTNP, only laser, and Chlorhexidine) are able to kill the bacteria successfully, and our developed treatment method was more than 99.99% effective in killing the *S*. *mutans* bacteria. To evaluate the effectiveness of laser treatment in situ, the viability of bacterial cells within treated biofilms was analyzed by confocal laser scanning microscopy (Figure 6C). As shown in Figure 6C, the biofilm bacteria were alive after treatment in control, PTNP-only, and laser-only groups. But the PTNP-with-laser treatment resulted in a highly effective method that rapidly killed most of the bacteria. We also noticed that, while the biofilm treated by chlorhexidine harbored predominantly dead bacteria in imaging, the bacteria recovered from the jaw were not significantly reduced. This may reflect differences in the location of the imaging analysis and recovery of plaque from both smooth and sulcal surfaces for the CFU counting. For confocal imaging, given the anatomical constraints of the jaw, we could only visualize the live/dead bacteria on smooth surface of the teeth, which could not depict the large number of live bacteria inside the sulcus that are more difficult to kill. Chlorhexidine, a highly cationic molecule, is known for its poor penetration into deeper biofilms. As a result, the bacteria in the deep layers (e.g., sulcal areas) might remain alive while most of the bacteria on smooth surfaces would be killed (3). For CFU counting, the jaws were subject to an optimized sonication procedure that recovers all the bacteria from both smooth and sulcal surfaces, which are harvested and counted. Despite technical differences, both confocal imaging and CFU counting clearly show that PTNP with laser was highly effective against the plaque bacteria compared with other conditions.

**Figure 7:**
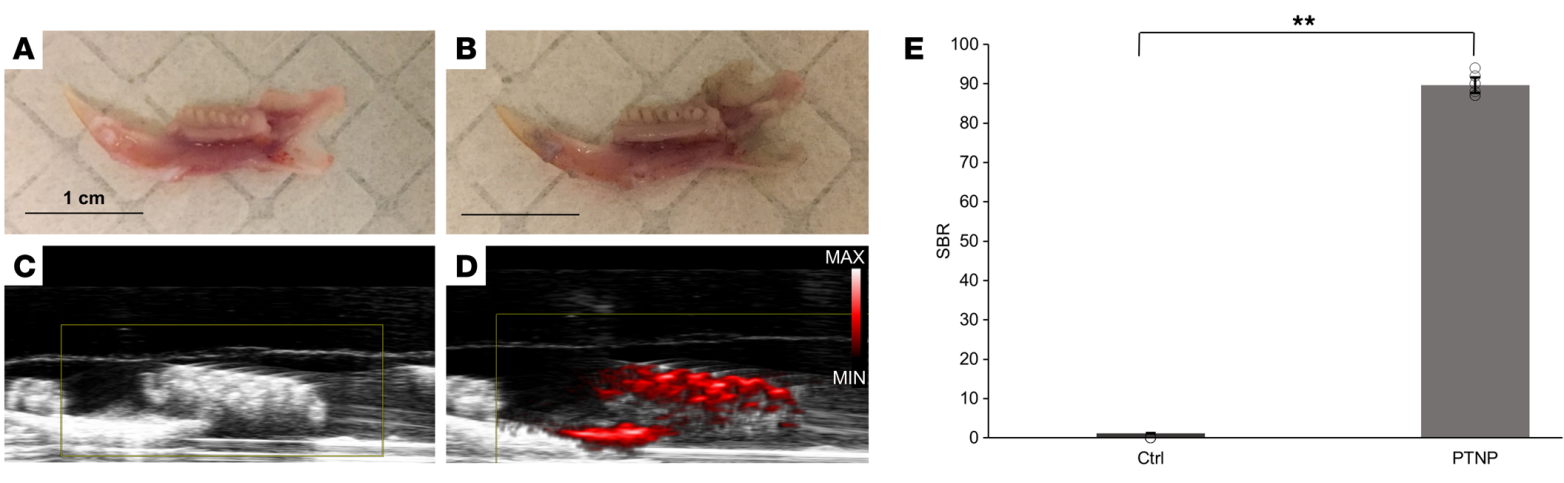
PTNP exhibit a strong PA signal in our oral model. Representative photographs of infected teeth (**A**) control and (**B**) incubated with PTNP (Scale bar: 1 cm). Grayscale ultrasound images overlaid with PA images of (**C**) control and (**D**) incubated with PTNP. (**E**) SBR of the infected teeth with and without PTNP (*n* = 6). Statistical analyses for the experimental data were performed using a mixed-model ANCOVA and SAS 9.5 (SAS Institute). **P < 0.005.

We next evaluated biofilm imaging and diagnostic potential of PTNP in our oral model. For the oral PA experiment, we imaged jaws incubated with either vehicle (a buffer or solvent as a control) or PTNP (Figure 7, A and B). The PA images reveal strong contrast for the teeth that had been treated with PTNP, while no signal was detected for the control group (Figure 7, C and D). PA image analysis showed that infected rat teeth that were incubated with PTNP had a 72-fold signal increase compared with the control (Figure 7E). PA has recently been used in oral applications in patients (44, 72) and these data confirm the infection on the teeth using PTNP and suggests that PA could also be used to diagnose oral infections.

### Treatment of Staphylococcus aureus–infected wounds using PTNP antibiofilm agents

**Figure 8:**
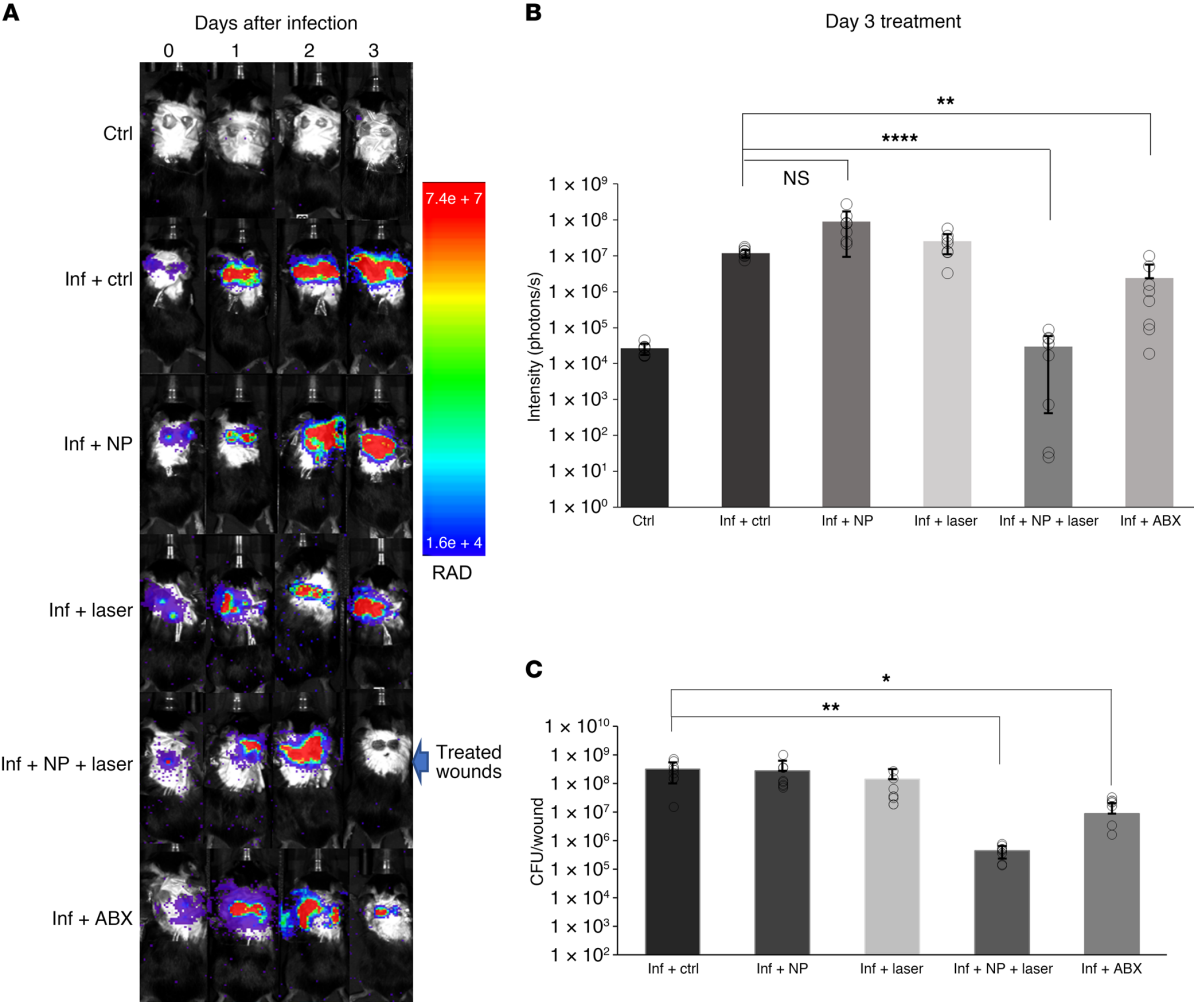
Bioluminescence in vivo imaging of an excisional wound infection model confirms the efficacy of PTNP. (**A**) BLI of mice infected with *S*. *aureus*. (**B**) Analysis of bioluminescence signal intensity after treatment. (**C**) The viability of *S*. *aureus* extracted from wounds treated as noted. In the graphs Ctrl, Inf, and NP denote control, infection, and PTNP (*n* = 8). The statistical analysis employed here involves the use of a 2-tailed *t* test and 2-way ANOVA with Weltch and Šidák’s multiple comparison adjustments. *P < 0.05, **P < 0.005, and ****P < 0.0005.

The feasibility of this treatment strategy to disinfect wounds was also investigated in vivo. A delayed inoculation *S*. *aureus* wound model was used to test the applicability of this technique (73, 74). As presented in Figure 8A, infection progress was monitored using bioluminescence imaging (BLI). The PTNP-with-laser group was the only group that showed no infections in the BLI images after 5 minutes of treatment (Figure 8B). To confirm the reliability of this technique, animals were euthanized, and wound tissues were extracted for bacterial plate counting (75–77).

Figure 8C shows the viability of *S. aureus* in our wound model for different treatment groups. Infected wounds that were treated with PTNP and laser irradiation had a remarkable decline in bacteria viability, in agreement with our in vitro studies. These results suggest that the treatment of the infected wounds with *S*. *aureus* bacteria using PTNP and laser killed most of the bacteria within a short time, however, the laser only, PTNP only, and topical gentamicin, an antibiotic positive control (ABX), did not have the same killing effect.

One of the greatest advantages of this method is to be able to control the heat generated by PTNP and monitor the infected wounds using an infrared thermographic camera. Here, the camera detected the surface temperature of tissues, treated, and nontreated wounds. We observed an increase in wound temperature for the groups treated with PTNP during laser irradiation (Supplemental Figure 13, A and B). However, ABX, control, laser, and PTNP-only treated mice showed the least heating effect, increasing approximately 5°C above mouse’s body temperature; this observation is in line with our in vitro data showing an increase in temperature of DI water when irradiated with NIR laser (Figure 3A). On the other hand, wound temperature increased by 20.3 ± 1.4°C when treated with PTNP and laser irradiation for 5 minutes (Supplemental Figure 13C). The topical treatment of infected wounds using this method provides the ability to control heat delivery at a local scale. As demonstrated in Figure 4, A and B, depending on the irradiation strategy used (i.e., a diffuse or focused laser beam), homogenous or localized biofilm treatment can be achieved that effectively kills bacteria without damage to the surrounding tissues in a short treatment duration. Here, we adjusted the NIR light to irradiate only the infected parts. This approach represents a potentially valuable treatment method in which PTNP can be employed for biofilm-associated infectious diseases treatment such as catheters, heart valves, and prosthetic joints.

We also examined our multi-functional nanoagent to image wound infections in vivo. To visualize the infected wounds, 20 μL of 2.5 mg/mL of PTNP solution was dropped onto the wounds area. Then, the PA imaging was performed after 10 minutes, while the mice were still under anesthesia. Similar to oral biofilms, PTNP-treated mice showed high signal intensity localized to the infectious wounds, while no signal was detected in the controls (Supplemental Figure 14).

## Discussion

Here, we have demonstrated that PTNP are effective theranostic antibiofilm agents, enabling rapid photoablation and biofilm disruption. We show the utility of approach in vitro, against 2 different bacterial species, and in a pair of in vivo preclinical models. This approach represents advancements over existing theranostic agents in demonstrating strong extinction spectra in the NIR region, excellent photothermal conversion efficiency, and capability in imaging of biofilm bacteria and eliminating them rapidly. A variety of gold nanostructures have been used for PTT previously, such nanorods and nanoshells. However, the synthesis of those structures typically involves the use of organic solvents or toxic chemicals, which require cumbersome procedures to eliminate or displace. The synthesis of PTNP, as described herein, avoids the use of such organic solvents and toxic reagents, with the primary reagents used being safe materials, i.e. sodium citrate and ascorbic acid. Therefore, the prospects for scale-up synthesis and clinical translation of PTNP are improved compared with nanorods and nanoshells (58, 59, 61).

PTNP demonstrate several remarkable features that are useful for the treatment of infectious diseases. First, it has a complex morphology and is nontoxic and biocompatible, which provides considerable interest for biomedical applications. Second, it can be used as a dual-use agent for both imaging and therapeutic applications to diagnose, degrade, and disinfect oral and skin biofilms. Third, it can be used in both wide-area irradiation and precise spatial control, which could provide homogenous or localized biofilm treatment to effectively target, activate, and kill bacteria. Fourth, this treatment method using PTNP is antibiotic free, and therefore there is no possibility of antibiotic resistance developing. Fifth, the biofilm killing using this approach is expeditious and low cost. We find that the amount of the bacteria decreases dramatically by using 3.75 mg of PTNP (when scaling to a human dose of 20 mL), which will only cost about $0.20. The other equipment that is needed to perform the biofilm treatment is a NIR laser that can be used for many years and costs around $200, which is an affordable expense for a clinical office. Alternatively, these agents could be used by patients at home, in combination with low cost, whole surface irradiators, which are already on the market. Our vision is that these PTNP could be incorporated into a variety of daily oral and skin health maintenance products, such as mouth rinses, gel strips, and photothermal bandages.

This study has some limitations. We have assessed biofilm killing but not therapeutic outcomes (e.g., preventing cavities or enhancing wound healing) so future studies will investigate whether PTNP can control cavities or accelerate wound healing. We tested our antibiofilm agent with a single wavelength NIR light. This can be fine-tuned further or tested on other light sources. Exploring drug loading or targeting within PTNP structures to either enhance antibiofilm efficacy or other functionalities (e.g., growth factors and antidemineralizing agents) could also lead to improved therapeutic outcomes. Moreover, the use of this treatment method and antibiofilm agent for other biofilm infections including those in catheters, heart valves, lungs, and prosthetic joints could be investigated.

In summary, dual purpose biocompatible PTNP structures, which have photothermal characteristics that detect, target, and kill bacteria within biofilms without the need for broad-spectrum antibiotics, is highly appealing for oral and skin infectious diseases. These properties allow the development of a range of different oral care products and wound disinfections. Here, we showed that this treatment method can accurately and selectively deliver the desired amount of heat to the infected area without damaging the healthy tissue and can be carried out in a very short time to target infected bacteria. The results of this research will advance the field of biofilm treatments by introducing new structures of antibiofilm agents that are activated under illumination by NIR light, resulting in biofilm disruption in vitro and in vivo in less than a minute.

## Methods

### Nanomaterial synthesis and characterization

PTNP were synthesized via the Turkevich method, a seeded growth method, and GRR using chloroauric acid (HAuCl_4_). In brief, 16 ± 2 nm seeds were prepared using the Turkevich method, and 78 nm cores were synthesized using the seeded growth method. 3 mL of 1 mM aqueous AgNO_3_ (Sigma-Aldrich) was reduced on the 78 ± 3 nm cores using 1 mL of 1 mM L-ascorbic acid (AA) (Thermo Fisher Scientific), making core-shell structures. Then, 10 mL of 500 μM aqueous HAuCl_4_ (Sigma-Aldrich), was added at a rate of 1 drop per second to create Au-Au core-cage structures, here termed PTNP (54). Ligand exchange was performed using 5 mL of 0.1 M DEX (Dextran T-10, Pharmacosmos). Next, the mixture of PTNP and DEX was allowed to stir overnight. The final, coated nanoparticles were then collected by centrifugation at 1200*g* for 10 minutes.

### UV-visible spectroscopy

UV-visible spectra were recorded using a UV-visible spectrophotometer (Thermo Fisher Scientific).

### Electron microscopy

Characterization of PTNP was done using a transmission electron microscope (JEOL 1010) operating at 80 kV and scanning electron microscope (SEM, Quanta 600 FEG, FEI). We examined PTNP binding using TEM and SEM to visualize and analyze PTNP distribution within biofilm ultrastructure (78). First, *S*. *aureus* biofilms treated either with PTNP or vehicle (Supplemental Figure 3) were washed with DI water to remove unbound materials. Biofilms were fixed with 2.5% glutaraldehyde and 2.0% paraformaldehyde in 0.1 M sodium cacodylate buffer, pH 7.4, overnight at 4°C. Prepared samples were fixed in 2.0% osmium tetroxide with 1.5% K_3_Fe (CN)_6_ for 1 hour at room temperature and rinsed in DI water. Then, samples were dried and were embedded in EMbed-812 (Electron Microscopy Sciences). Several 0.5 μm semithin sections were cut into the sample for prereview before cutting 70 nm thin sections for TEM. 1% uranyl acetate and SATO lead were used to stain the thin sections of the final samples. TEM images were acquired using a JEOL 1010 electron microscope fitted with a Hamamatsu digital camera and AMT Advantage NanoSprint500 software. EDS was also performed to detect gold and silver from PTNP, with corresponding elemental mapping.

### Hydrodynamic diameter and ζ potential measurements

Hydrodynamic diameter and ζ potential of PTNP were examined using dynamic and electrophoretic light scattering (Nano ZS-90 Zeta sizer). All DLS data were acquired by diluting 100 μL of 0.1 mg/mL PTNP structures to 1 mL with DI water.

### Inductively coupled plasma optical emission spectroscopy

PTNP concentrations were measured using inductively coupled plasma optical emission spectroscopy (ICP-OES, Spectro Analytical Instruments GmbH) to measure the gold and silver concentrations in PTNP samples and also within tissue samples according to a protocol published elsewhere (54).

### In vitro heating

The photothermal properties of PTNP were measured using NIR laser irradiation to induce heating of these structures. A milliliter of PTNP (10 μg/mL) was added to a 2 mL quartz cuvette, and a fiber optic thermometer (Nomad, Qualitrol-Neoptix) was inserted 5 mm below the liquid surface. Solutions were then irradiated with an 808 nm laser (OEM Laser Systems) at 2 W/cm^2^ power for 10 minutes. Temperature was recorded every 10 seconds. DI water was used with the same procedure as a control. For the laser power and PTNP concentration dependency experiment, the laser was turned off after reaching a constant temperature (i.e., after 10 minutes). The photostability, shown in Supplemental Figure 5, was measured at 10 μg/mL PTNP and with NIR laser irradiation with 2 W/cm^2^. The laser was turned off after reaching a plateau in temperature and the sample was allowed to cool down to the room temperature before the laser was turned on again to reach the maximum temperature for the next round. The final temperature is given as a Δ to exclude minor variations in baseline temperature and to highlight the differences in temperature gain caused by the conditions used. This experiment was repeated 5 times. All experiments were performed at room temperature with ambient light.

### Laser Set-up for in vitro and in vivo experiments

Irradiation using collimated laser beam (Precise spatial control mode): The experiments in which light was collimated and the flux was precisely controlled have been carried out using NIR laser (808 nm, OEM Laser Systems, flux 0.5 or 2 W/cm^2^). The beam had a rectangular cross-section (2mm × 8mm), and it could cover all of the teeth of a rat. The object was irradiated during different time periods (15 or 30 seconds, or 1, 5, and 10 minutes). This approach can be clinically translated and used for treatment of teeth cavities and biofilm-forming infections in humans.

### Wide-area irradiation

The same laser coupled to a fiber (808 nm, OEM Laser Systems) could be used for wide-area irradiation. The beam was focused on the entrance aperture of a multimode glass fiber (Dolan Jenner, 1 m, 4 mm diameter) using a lens (f = 5.5 mcm) mounted on a 1D translation stage (Thorlabs) such that the size of the spot on the fiber aperture was approximately 2–3 mm in diameter. Such slight defocusing allowed us to avoid overly high fluxes at the aperture and prevent overheating. The light emerged from the other end of the fiber uncollimated, and therefore by adjusting the distance between the fiber tip and the object, round areas of different diameter could be irradiated. The photon flux across the area was approximately uniform, as judged by visual examination, and it was calculated from the laser power and measured by an optical power meter (Thorlabs) immediately after the fiber and the area diameter.

### In vitro oral biofilm model

*Streptococcus mutans* (*S. mutans*) UA159 (ATCC 700610) was used as an oral biofilm bacterium and was grown in ultra filtered (10 kDa molecular-mass cutoﬀ) tryptone-yeast extract broth (UFTYE; 2.5% tryptone and 1.5% yeast extract) containing 1% (wt/vol) glucose at 37°C and 5% CO_2_ to midexponential phase. *S*. *mutans* biofilms were formed on sHA discs with surface area of 2.7 ± 0.2 cm^2^ (Clarkson Chromatography Products Inc.), as described previously (75). Each HA disc was coated with filter-sterilized saliva for 1 hour at 37 °C and the saliva was prepared as described elsewhere (79). The discs were vertically suspended in 24-well plates using a customized wire disc holder that mimics the smooth surfaces of the tooth. These sHA discs were each inoculated with approximately 2 × 10^5^ CFU of *S*. *mutans* per milliliter in UFTYE culture medium (pH 7.0) containing 1% (w/v) sucrose at 37°C with 5% CO_2_. The culture medium was changed at 19 and 29 hours (twice daily) until 43 hours. Then, the biofilms were collected and analyzed for PTNP binding, biomass reduction, and bacterial killing, as described below.

### Bacterial killing and biomass reduction by PTNP with laser irradiation

To assess the antibiofilm effect of PTNP within biofilms, the bacteria were grown overnight and then 1:100 dilute from overnight culture were added into fresh medium for biofilm assay. For the skin biofilm formation, *S*. *aureus* isolates (USA300, Newman 502A) were grown at 37°C in liquid luria broth (Thermo Fisher Scientific) at 300 rpm. Then 100 μL of the dilution was added per well in a 96 well dish and the plates were incubated at 37°C with 5% CO_2_ for 24 hours. Then the plates were washed 3 times with DI water before incubating with different concentrations of PTNP (0, 0.1, 0.25, and 0.5 mg/mL) for another 24 hours. For oral biofilm formation, the *S*. *mutans* biofilms were grown on sHA discs as described above. The sHA discs and biofilms were topically treated twice daily by placing them in 2.8 mL of PTNP (at either 0.12 or 0.25 mg/mL) in 0.1 M NaAc (pH 4.5) for 10 minutes at room temperature at specific time points indicated in Supplemental Figure 2. After incubating the discs with PTNP, all the discs, both controls and PTNP samples, were inserted in DI water 3 times and washed out completely. At the end of the experiment, the PTNP-treated biofilms were exposed to an NIR laser (808 nm, OEM Laser Systems, 808 nm at a power of 0.5 and 2 W/cm^2^) for different time periods (15 seconds, 30 seconds, 1 minute, 5 minutes, and 10 minutes). After NIR laser exposure, some samples were used for visualization and confocal microscopy as described below, and some were used for CFU counting and dry weight assay. For the latter purposes, the biofilms were washed with sterile saline solution (0.89% NaCl) 3 times and removed by a spatula from sHA discs. Then, the samples were homogenized using bath sonication (80, 81). Samples of these biofilm suspensions were diluted in different concentrations and plated onto blood agar plates. Then, these plates were placed in an incubator at 37°C with 5% CO_2_ for 48 hours. The total numbers of viable cells in each biofilm control and treated biofilm were determined by counting CFU. The remaining suspension was centrifuged at 5,500*g* for 10 minutes and washed with DI water twice and dried in oven at 90°C for 2 hours. Lastly, the final residue was weighed to assess biomass reduction (13, 80).

### Dynamics of bacterial killing of PTNP within intact biofilm and EPS structures

The distribution of PTNP, the bacterial killing effect of PTNP, and EPS degradation within biofilm were visualized using a confocal ﬂuorescence microscope (LSM 800, Zeiss) with a ×20 (numerical aperture, 1.0) water immersion objective. Live and dead cells were labeled using SYTO 9 (485/498 nm; Molecular Probes) and propidium iodide (PI, 535/617 nm; Molecular Probes), and Alexa Fluor 647-dextran conjugate (647/668 nm; Molecular Probes) was used for labeling EPS.

### Cell viability

BJ5ta (human fibroblast) and C2BBe1 (human colorectal adenocarcinoma, epithelial) cells were purchased from ATCC. Primary human gingival epithelial cells and HaCaT were donated by Manju Benakanakere and John Seykora (both of the University of Pennsylvania, Philadelphia, Pennsylvania, USA), respectively. The effect on cell viability of PTNP was assessed using the MTS [(3-(4,5-dimethylthiazol-2-yl)-5-(3-carboxymethoxyphenyl)-2-(4-sulfophenyl)-2H-tetrazolium)] assay (CellTiter 96 cell proliferation assay kit; Promega) (82). Dulbecco’s modified eagle’s medium (DMEM) and medium 199, 10% FBS (Gibco) and 0.01 mg/mL of hygromycin B (Sigma-Aldrich) were used as a culture medium for BJ5ta cells. C2BBe1 cells were also cultured in a medium of DMEM, 10% FBS, 45 IU/mL penicillin and 45 IU/mL streptomycin (Gibco), with 10 μg/mL human transferrin (Sigma-Aldrich). Primary human gingival epithelial cells were cultured in keratinocyte serum-free medium (Invitrogen) and HaCaT cells were cultured in DMEM with Glutamax,4.5 g/L D-glucose, 110 mg/L Na Pyruvate (Gibco/Invitrogen #10569), 5% FBS and mixed with antibiotics streptomycin (100 μg/mL) and penicillin (100 U/mL). The cells were grown at 37°C in a 5% CO_2_ humidified incubator. To determine the cytotoxicity of PTNP, 1 × 10^4^ cells per well were seeded in 96-well plates, and then the plates were incubated at 37°C in a 5% CO_2_ atmosphere for 4 hours (the cell viability of HaCaT cells were also measured after 24, 48, and 72 hour incubation with PTNP). Then, the media was carefully removed, and the cells were washed gently with sterile PBS twice and replaced with fresh media containing PTNP at different concentrations. The plates were then incubated at 37°C in 5% CO_2_ atmosphere for different times (24, 48, and 72 hours). After this incubation, the media was removed, the cells were washed with PBS, then 20 μL MTS reagent and 100 μL cell culture medium was added to each well. The plates were incubated at 37°C in a 5% CO_2_ atmosphere for 1 hour, then the absorbance was measured at 490 nm using a plate reader. The percentage of relative cytotoxicity of each concentration of PTNP was calculated compared with control and the data presented as mean ± SD (*n* = 3).

The effect of PTNP on cell viability was also evaluated while the cells were irradiated with the NIR laser. In these experiments, HaCaT cells were plated in 24-well plates (10,000 cells per well) and incubated at 37 °C in a 5% CO_2_ atmosphere for 24 hours. Then, the cells were washed with PBS and exchanged with the fresh media containing PTNP with different concentrations (*n* = 3 wells per condition). The following day, the cells were washed with PBS twice and replaced with fresh media. Immediately after media exchange, wells were irradiated with a NIR laser (808 nm wavelength, 0.25 and 0.5 W/cm^2^) for 7 minutes. Plates were then incubated at 37°C in a 5% CO_2_ atmosphere to be analyzed for laser cytotoxicity of cells at 2, 24, 48, and 72 hours after laser irradiation using the live-dead assay. The media was exchanged with the live-dead reagents consisting of 2 mL PBS, 2 μL ethidium-1 homodimer for dead cells (used from as received stock), and 0.5 μL of stock Calcein AM for live cells. After 20 minutes incubation with the live-dead cocktail, 6 micrographs per well were taken using a Nikon Eclipse Ti–U fluorescence microscope. The images were acquired using 495/515 nm filter for Calcein and 528/617 nm filter for Ethidium Homodimer-1 (EthD-1). The number of live or dead cells were counted in each image to analyze cell cytotoxicity after laser irradiation at different time points.

### In vitro PA imaging

A Vevo Lazr device (VisualSonics) with LZ550 transducer were used to acquire the photoacoustic images. Twenty μL of PTNP were inserted into 0.5 mm diameter polyethylene tubing which was then covered in 2 to 3 cm of water. PTNP were prepared at different PTNP concentrations (0, 0.06, 0.12, 0.25, and 0.5 mg/mL). The transducer distance from sample was 1.1 to 1.5 cm.

The PA imaging of PTNP within skin biofilms was also carried out using the Vevo Lazer device and LZ550 transducer. The *S*. *aureus* bacteria were cultured in 96 wells using Bacto Tryptic Soy Broth (TSB) (BD Biosciences) as a growth media. After 24 hours, the biofilm bacteria were incubated with different PTNP concentrations (0, 0.12, 0.25, and 0.5 mg/mL) for another 24 hours. Then, the biofilm treated with PTNP was washed 3 times and was covered in 2 to 3 cm of water. The ultrasound gain and PA gain were adjusted at +27 dB and 22 dB, respectively.

### Animal and infectious disease models

#### Ex vivo efficacy of PTNP for oral biofilms.

An established rodent model that mimics dental caries, including *S*. *mutans* infection of rat pups, was used for ex vivo experiments for oral infectious disease (80, 83). Briefly, 15-day-old female Sprague−Dawley rat pups were purchased from Harlan Laboratories. First, the animals were infected orally with actively growing (midlogarithmic) culture of *S*. *mutans* UA159, and their infection was confirmed via oral swabbing. The animals’ diet was based on the NIH cariogenic diet 2000 (TestDiet) and 5% sucrose water ad libitum. The animals were categorized into 5 treatment groups. The treatment groups were: (a) control (0.1 M NaAc buffer, pH 4.5), (b) PTNP only (0.25 mg/mL), (c) laser only, (d) PTNP and laser irradiation, and (e) chlorhexidine. The animals’ physical appearance was monitored daily, and the body weights were recorded weekly. The experiment proceeded for 3 weeks (21 days). At the end of the experimental period, the animals were sacrificed and their jaws were surgically removed and aseptically dissected. Jaws with infected teeth were placed in a solution of PTNP (at 0.25 mg/mL), chlorhexidine or vehicle for 10 minutes and washed in DI water (the jaw was dipped in DI water 3 times), which was immediately followed either by NIR laser irradiation (at 808 nm wavelength and 2 W/cm^2^) for 1 minute or mock irradiation. Some jaws were used for PA imaging and others were used for CFU counting and biofilm biochemical assessment. For the latter, the plaque−biofilm samples were removed from the jaws and teeth using sonication and prepared for analysis (83).

### In vivo efficacy of PTNP in skin biofilm

To test the ability to kill bacteria in vivo, a modified *S*. *aureus* (Xen36 luminescent strain, a pathogen known to cause wound infections) delayed-inoculation wound infection model and surgery protocols were performed as described previously (74, 77). Male 8-to-12-week-old C57BL/6J mice were bred in the vivarium at Stanford University. The ones that underwent surgery received additional Supplical Pet Gel (Henry Schein Animal Health). Mice were divided into 5 groups: (a) control, (b) PTNP only, (c) laser only, (d) PTNP + laser, and (e) topical antibiotic positive control (gentamicin). 24 hours after surgery, 1-to-2 × 10^8^ CFU of XEN36 bioluminescent strain *S*. *aureus* were injected through the transparent film dressing into each wound. For the subsequent 48 hours, mice were monitored at least daily for wound condition and signs of distress. Meanwhile, the infected wounds were monitored with BLI to visualize the infection rate in each wound. After 48 hours, the treatment procedures were carried out. For the PTNP-only and PTNP + laser groups, 20 μL of 0.25 mg/mL of nanoparticles were applied to an inoculated skin wound. After an incubation time of 10 minutes, while the mice were under anesthesia, the infected wound with the associated nanoparticles,and laser only group were exposed to the laser treatment (808 nm NIR laser, at 0.7 W/cm^2^) for a duration of 5 minutes. Other groups were treated with the same condition, but without the NIR laser irradiation. Animals were euthanized after the treatment and in vivo imaging. The treated and untreated wounds were extracted for bacterial plate count. The surface temperature of wounded mice was also monitored before treatment, after 1, 2, 3, 4, and 5 minutes of treatment using an FLIR 1 thermal imaging camera (FLIR Systems).

### In vivo imaging

#### BLI.

BLI was performed by using a Lago X imaging system (Spectral Instruments Imaging). Mice infected with luciferase-expressing XEN36 *S*. *aureus* were anesthetized with 2.5% isoflurane, and bioluminescent signals were quantified by using Aura software (Spectral Instruments Imaging). The acquisition parameters were adjusted as follows; exposure time 30 seconds, binning low (2), f/stop 1.2, and FOV 25. The luminescence data was analyzed using Aura software.

#### In vivo PA imaging.

PA was performed with a VisualSonics Vevo 2100/LAZR high-frequency ultrasound/PA scanner with 256 array transducers. The experiment was carried out with skin infected male 8–12-week-old C57BL/6J mice (*n* = 3). The procedure was performed under anesthesia with isoflurane (1.8%–3%, 2 L/min O^2^) before imaging on control and PTNP treated mice (20 μL of 0.25 mg/mL) for 10 minutes. The images were acquired using 800 nm NIR light, with ultrasound gain of +27 dB, PA gain 35 dB, and priority 95%.

### Ex vivo PA imaging

Ex vivo PA imaging was captured using a Vevo Lazr @2100 device (VisualSonics). PA imaging was performed on infected jaws (*n* = 3), which were exposed to PTNP (0.25 mg/mL) for 10 minutes, and infected jaws, which had no incubation with PTNP (control). The jaws were adhered to a dish with glue and were covered with 1–2 cm water. An LZ550 transducer, 800 nm NIR light, with ultrasound gain of +27 dB, and PA gain 35 dB, were used to acquire PA images. Image analyses were performed by placing ROIs on the images of control and biofilms incubated with PTNP. Then, signal-to-background ratio (SBR) values were calculated from these measurements (from 6 images per group).

### Statistics

Statistical analyses for the experimental data were performed using a 2-tailed *t* test, mixed-model analysis of covariance (ANCOVA), and SAS 9.5 (SAS Institute). For the in vivo experiments, an analysis of outcome measures was done with transformed values of the measures to stabilize variances. The data were then subjected to ANOVA for all sets of data. *P* values of less than 0.05 were considered statistically significant.

### Study approval

The reported studies in animals were approved by the Stanford University Institutional Animal Care and Use Committee (Palo Alto, California, USA) and the University of Pennsylvania Institutional Animal Care and Use Committee (Philadelphia, Pennsylvania, USA).

### Data availability

The underlying data from the manuscript is available from the corresponding author upon request. Values for all data points in graphs are reported in the Supporting Data Values file.

## Author contributions

MH, DPC, HK, and PLB designed the research study. MH, CRV, ZR, YLiu, YH, YLi conducted the research study. MH, JCH, AA, YCD, SK, PJ, and AZ acquired and analyzed the data. MH, EG, PLB, HK, DPC participated in editing the manuscript.

## Supplementary Material

Supplemental data

Supporting data values

## Version 1 08/31/2023

In-Press Preview

## Version 2 11/01/2023

Electronic publication
